# Counselling Patients with Uterine Fibroids: A Review of the Management and Complications

**DOI:** 10.1155/2012/539365

**Published:** 2012-01-09

**Authors:** Donnette Simms-Stewart, Horace Fletcher

**Affiliations:** Department of Obstetrics and Gynaecology, Faculty of Medical Sciences, The University of the West Indies, Mona, Kingston, Jamaica

## Abstract

Fibroids are very common in Afro-Caribbean women. They can cause severe complications. The treatment modalities are not without risk and should be weighed against the complications of the fibroids.

## 1. Introduction

Leiomyomata uteri (uterine fibroids) are benign tumours of the smooth muscle of the uterus. These tumours have prevalence ranging from 20 to 50% of women depending on the age, ethnicity, parity, and methods use to assess their presence. In one series, they were said to be present in 77% of postmortem specimens where detailed examination of the uterus was done looking for theses fibroids. In that series, over 50% of the women were asymptomatic [1].

Uterine fibroids grow under the influence of the hormone oestrogen and are most often seen after the menarche, and tend to shrink after the menopause. Typically the patient is nulliparous or of low parity and they are more commonly seen in women of African ancestry.

Common complications of uterine fibroids include menorrhagia with symptoms of anaemia, dysmenorrhoea, pressure symptoms, abdominal distension, and infertility. Infertility appears to be an incidental finding rather than a consequence of the fibroid, except in cases of submucosal fibroids [2, 3]. Other complications include degeneration, torsion, prolapse of a submucous fibroid, ureteric obstruction, venous thromboembolism, intestinal obstruction, and malignant transformation.

## 2. Menorrhagia

Menorrhagia is defined as regular, cyclic menstrual flow with excessive volume and durations. Clinically, menorrhagia is defined as total blood loss exceeding 80 mL per cycle or menses lasting longer than 7 days [4]. In practice, measuring menstrual blood loss is difficult. Thus, the diagnosis is usually based upon the patient's history. These patients will complain of flooding, overflow, and passage of large clots. In many cases women will notice that they have an increased need for sanitary napkins compared to those obtained in the past.

Menorrhagia must be distinguished clinically from other common gynecologic diagnoses. These include metrorrhagia (flow at irregular intervals), metromenorrhagia (frequent, excessive flow), and polymenorrhea (bleeding at intervals <21 d).

The mechanism by which fibroids cause menorrhagia is not well understood. The blood supply to the fibroid is different compared to the surrounding endometrium and is thought to function independently. This blood supply is greater than the endometrial supply and may have impeded venous return, causing pooling in the areas of the fibroid. Heavy pooling is thought to weaken the endometrium in that area, and breakthrough bleeding ensues.

Fibroids located within the uterine wall may inhibit muscle contracture, thereby preventing normal uterine attempts at haemostasis. In many cases the fibroids increase the size and volume of the uterus and uterine lining with increased bleeding [5].

### 2.1. Intermenstrual Bleeding

Submucosal fibroids may also present with intermenstrual bleeding. This is especially prevalent with prolapsed submucous fibroids. Any woman with fibroids and intermenstrual bleeding must, however, have a pelvic examination and pap smear to make sure an obvious cervical cancer is ruled out.

### 2.2. Haematological Disorders

This is most commonly iron deficiency anaemia secondary to uterine haemorrhage. However women with fibroids sometimes have polycythemia [6] due to increased production of erythropoietin and also thrombocytosis in response to excess bleeding [7]. These two hematological complications are well known to be associated with venous thromboembolism and are one mechanism by which this occurs in patients with fibroids.

### 2.3. Pressure Effects

These are usually manifest on the urinary tract (distorting the bladder producing urinary frequency or paradoxically acute retention), ureters (causing hydroureters and hydronephrosis), rectum (causing tenesmus), and veins (principally the left common iliac vein causing varicosities, venous thromboembolism [8, 9], and leg oedema. These problems are more likely with large fibroids and renal and venous obstruction are potentially life threatening. Women diagnosed with these problems need to have the fibroids removed to prevent permanent kidney damage of pulmonary embolism.

### 2.4. Pain

Women with uterine fibroids typically have spasmodic dysmenorrhoea [10], with the uterus going into spasms as it tries to expel the large clots and excess blood. The pain typically starts with the bleeding and ends abruptly with the end of the bleeding. This must be differentiated from congestive dysmenorrhoea which occurs with conditions such as endometriosis where the pain starts before any bleeding and continues for several days after the end of bleeding. Many women have both conditions so women who have clinically palpable fibroids but have congestive-type dysmenorrhoea quite often have endometriosis at surgery.

Infarction in fibroids (spontaneous infarction, torsion causing infarction) can cause quite severe acute pain. Pedunculated subserosal or submucosal fibroids can undergo torsion. Extrusion of a submucosal fibroid polyp may be associated with “labour-like” pains.

Fibroids may enlarge to the point that they outgrow their blood supply and undergo necrosis (red degeneration). This also causes a great deal of pain for patients. This most commonly seen in pregnant women with fibroids and is a common problem in these women.

### 2.5. Degeneration in Fibroids

There are several types of degeneration in a fibroid [11]. *Hyaline* changes is the commonest; it is present in two-thirds of fibroids and consists of deposition of mucopolysaccharide around the muscle fibres. *Calcification* is also common, especially after the menopause. These calcifications have been known to result in intestinal obstruction in postmenopausal women [12]. *Fatty changes* are uncommon and usually asymptomatic. *Red degeneration* (infarction of fibroid) is commoner in pregnancy.

### 2.6. Infection

Infection with pyometra may also be associated with submucosal fibroids. It is most likely to occur in the puerperium during uterine involution and when the cavity is colonized by microorganisms. Pyomyoma is a rare condition which is seen in pregnant women and perimenopausal women with vascular disease [8, 9]. In these women the fibroid becomes necrosed and becomes infected. The triad of (1) bacteremia or sepsis (2) leiomyoma uteri; and (3) no other apparent source of infection should suggest the diagnosis of pyomyoma [13].

### 2.7. Complication in Pregnancy

Red degeneration is the most common problem with fibroids in pregnancy and usually causes severe pain. These women should be treated conservatively as any surgical procedure during the pregnancy can result in preterm delivery and foetal loss. Although myomectomy in early pregnancy has been successfully done, it is not recommended because of the risk of maternal haemorrhage and foetal loss. Other associated problems include placenta praevia and intrauterine growth restriction [14], fetal obstruction with malpresentation or obstructed labour, postpartum haemorrhage, and puerperal infection. Women with fibroids who get pregnant should be allowed to continue pregnancy and all efforts made to make sure they have a normal haemoglobin at delivery. Delivery should be planned to ensure the correct route is chosen. Vaginal delivery is preferred if feasible. Caesarean section is done when needed and usually a lower segment procedure is better. If small fibroids are in the lower segment then these can be removed at the same time. If the lower segment has very large fibroids then a classical incision may be the better option. Caesarean myomectomy is a feasible option but with caution. The baby must be removed before myomectomy is done. If there is much bleeding during delivery the baby and the placenta, myomectomy must be postponed. An oxytocic agent must be used to prevent haemorrhage at myomectomy, and the use of vasopressin is a valuable adjunct [15]. Newer drugs such as carbetocin may be also very valuable here as the oxytocic effect lasts much longer than oxytocin [16]. In some women who have very large fibroids and have completed their family a subtotal hysterectomy at caesarean section may be of value to avoid an interval procedure.

### 2.8. Malignant Transformation

One of the most feared complications of fibroids by patients is if the fibroids can become cancerous.

The answer to this is that this is possible; however, these events are very rare occurring in less than 1% of patients with fibroids. Patients who have fibroids who have sudden rapid growth in size or women who notice other symptoms such as shortness of breath or increased abdominal discomfort need evaluation. CT scans and MRI can show atypical myomata and surgery may be recommended. However, the only definitive way to diagnose leiomyosarcomatous change is by histological examination.

Other rare histological types have been found such as intravascular leiomyomatosis and even the more rare intravascular leiomyosarcomatosis first described by Coard and Fletcher [17].

Perimenopausal and postmenopausal women who have large fibroids should be monitored for increased symptoms and have hysterectomy in the event that there is a suspicion of malignancy.

### 2.9. Infertility

Several studies have shown that submucous fibroids are associated with infertility, probably as a result of decreased implantation [14]. Some studies have also shown that submucous fibroids are associated with recurrent spontaneous abortions. However, in many cases the infertility preceded the fibroids and the fibroids have grown because of incessant ovulation. In many of these women the cause of the infertility is tubal occlusion or endometriosis. There may also be abnormalities of tubal motility or tubal obstruction based on the location of the fibroid. Otherwise fibroid is not a significant cause of infertility. Women who have infertility and fibroids should be properly evaluated with hysterosalpingograms and laparoscopy. Submucous fibroids if found should be removed by hysteroscopy [18]. Tubal surgery and myomectomy are notoriously inefficient in treating infertility as adhesion formation is inevitable. Fertility after one myomectomy is about 50% and after two myomectomies is only 15% [19]. The use of adhesion barriers may be beneficial but many of these have not had the benefit of randomized placebo-controlled trials.

## 3. Management

Management of a patient with uterine fibroids is highly dependent on the presentation and patient wishes. Other causes of abnormal bleeding need to be ruled out. In many cases the management of the fibroids is also risky and in some women the fibroids are best left alone.

Fibroids are very common and women with small fibroids who are asymptomatic are best left untreated. Women with symptoms who have small fibroids but are close to the menopause or who are trying to conceive should be treated conservatively with analgesics and hematinics. Women who have severe symptoms or very large fibroids usually need surgical intervention. This may be conservative with myomectomy done by laparotomy (all fibroids), laparoscopy (subserous fibroids), or hysteroscopy (submucous fibroids). All women going for myomectomy must also be consented for hysterectomy as haemorrhage is the main complication and hysterectomy may be life saving when done early enough.

Prior to any treatment women need proper evaluation with history and examination. This usually will give an idea of the severity of the condition. Women must also have proper laboratory investigation to confirm the severity of their condition.

A general history must be done to rule out other causes of bleeding since fibroids are so common. Some women may in fact be discovered to have a bleeding condition for the first time based on history. They must be asked about social habits such as excess alcohol intake leading to liver disease. They should also be asked about other factors suggesting bleeding diatheses such as bleeding from other orifices and also presence of purpura and eccymoses.

May women will have other complaints such as pica or shortness of breath suggesting anaemia. However, if this is an acute problem remember to rule out pulmonary embolism.

The examination findings must also be general before targeting the abdomen. Pallor abdominal swelling and pedal oedema are common findings. Unilateral swelling of the legs is a very suspicious complaint and DVT must be ruled out.

### 3.1. Laboratory Studies

A complete blood count (CBC) may be used as a baseline for hemoglobin and hematocrit or to rule out anemia polycythemia or thrombocytosis. The platelet count in conjunction with a peripheral smear may indicate thrombocytopenia, confirming a bleeding disorder in some cases.

**Iron Studies**

Total iron-binding capacity (TIBC) ferritin levels are used to assess iron stores when iron deficiency is found. This is not always needed and is done when there is any doubt about the type of microcytic anemia found.

When bleeding disorders are suspected, studies are used to rule out von Willebrand's disease, platelet disorders, and factor II, V, VII, or IX deficiency. These tests should be ordered sparingly because they are expensive tests for rare disorders usually done based on level of suspicion from the history and examination.

Pregnancy remains the most common cause of abnormal uterine bleeding in patients of reproductive age. Bleeding usually denotes threatened abortion, incomplete abortion, or ectopic pregnancy. A pregnancy test may be of value in cases where pregnancy is suspected as a possible cause of bleeding or pain.

Hormonal tests such as follicle stimulating hormone, luteinizing hormone, progesterone, thyroid function tests and prolactin level are tests done to rule out conditions that can cause ovarian dysfunction leading to possible menorrhagia.

Liver function and/or renal function tests are done when liver disease is suspected, such as in persons with alcoholism or hepatitis. It is very important to rule this out as a cause of bleeding prior to attempting surgery.

Urea and creatinine tests assess renal function especially if obstruction has been found on imaging.

### 3.2. Imaging Studies

Pelvic ultrasound is the best noninvasive imaging study to assess uterine shape, size, and contour; endometrial thickness; adnexal areas. It is also useful to evaluate the urinary system. Hydroureters and hydronephrosis are common findings in patients with fibroids an indication that surgical intervention is needed

Sonohysterography (saline-infusion sonography) where fluid infused into the endometrial cavity enhances intrauterine evaluation. One advantage is the ability to differentiate polyps from submucous fibroids.

### 3.3. Surgical Evaluation

Evaluation of the patient may require hysteroscopy in some cases and laparoscopy in others. In one series from Jamaica fibroids were found in 30% of patients who had hysteroscopy for abnormal uterine bleeding [20].

## 4. Treatment

### 4.1. Medical Care

Care should be tailored to the individual. Factors taken into consideration when selecting the appropriate treatment include the patient's age, coexisting medical diseases, family history, and desire for fertility. Medication cost and adverse effects are also considered because they may play a direct role in patient compliance.

Nonsteroidal anti-inflammatory drugs (NSAIDs) are the first-line medical therapy in ovulatory menorrhagia. These drugs have been found to be better than placebo in reducing menstrual blood flow [21]. 

NSAIDs reduce prostaglandin levels by inhibiting cyclooxygenase and increasing the ratio of prostacyclin to thromboxane. NSAIDs are ingested for only 5 days of the entire cycle, limiting their most common adverse effect of stomach upset and the risk of stomach ulceration.

Oral contraceptive pills (OCPs) are a popular first-line therapy for women who desire contraception.

Menstrual blood loss is reduced as effectively as NSAID's secondary to endometrial atrophy [22].

 OCPs suppress pituitary gonadotropin release, preventing ovulation. Common adverse effects include breast tenderness, breakthrough bleeding, nausea, and, possibly, related weight gain in some individuals. Care should be taken in use of OCPs in women with fibroids as these increase the risk of venous thromboembolism [23].

Gonadotropin-releasing hormone agonists are used on a short-term basis due to high costs and severe adverse effects. The agonists are effective in reducing menstrual blood flow. They inhibit pituitary release of FSH and LH, resulting in hypogonadism. A prolonged hypoestrogenic state leads to bone demineralization and reduction of high-density lipoprotein (HDL) cholesterol.

 Progestin therapy is the most frequently prescribed medicine for menorrhagia. Therapy with progestin results in a significant reduction in menstrual blood flow when used alone.

Progestin works as an antiestrogen by minimizing the effects of estrogen on target cells, thereby maintaining the endometrium in a state of downregulation.

Common adverse effects include weight gain, headaches, edema, and depression.

 Levonorgestrel intrauterine system (LIS) reduces menstrual blood loss by as much as 97% [24]. This is comparable to transcervical resection of the endometrium for reduction of menstrual bleeding [25].

The United States FDA approved a new indication for the levonorgestrel intrauterine system, for the treatment of menorrhagia in women who use intrauterine conception. Approval was granted subsequent to a randomized, open-label, active-control (medroxyprogesterone) clinical trial of women (*n* = 160) with established heavy menstrual bleeding. The results demonstrated that LIS reduced menstrual blood loss significantly compared with medroxyprogesterone (*P* < 0.001) [26]. Adverse effects of LIS include uterine bleeding or spotting, headache, ovarian cysts, vaginitis, dysmenorrhea, and breast tenderness.

Depo-medroxyprogesterone acetate (DMPA) which is inexpensive has been found in our unit to be very valuable, reducing menstrual bleeding and allowing women to improve their haemoglobin prior to surgery [27]. However like GnRh, DMPA is also antiestrogen and can decrease bone density when used for a long time [28].

 Danazol competes with androgen and progesterone at the receptor level, causing amenorrhea in 4–6 weeks. Androgenic effects cause acne, decreasing breast size, and, rarely, lower voice.

Tranexamic acid was the first nonhormonal product approved by the US FDA (in November of 2009) [29] for the treatment of heavy menstrual bleeding. Tranexamic acid is a synthetic derivative of lysine that uses antifibrinolytic effects by inhibiting the activation of plasminogen to plasmin.

The mechanism of action in treating heavy menstrual bleeding is by prevention of fibrinolysis and the breakdown of clots via inhibiting endometrial plasminogen activator.

In a recent, double-blind, placebo-controlled study, women taking 3.9 g/d of tranexamic acid showed a significant reduction in menstrual blood loss and an increase in their health-related quality of life compared with those taking placebo [30]. Common adverse effects include menstrual discomfort, headache, and back pain.

### 4.2. Surgical Care

Surgical management has been the standard of treatment for fibroids especially when medical therapy fails to alleviate symptoms. Surgical treatment ranges from a simple D&C to a full hysterectomy.

### 4.3. Dilatation and Curettage

A D&C should be used for diagnostic purposes. It is best done in women who are perimenopausal or any woman found with an excessively thickened endometrium on ultrasonography. It is not used for treatment because it provides only short-term relief, typically 1-2 months.

This procedure is used best in conjunction with hysteroscopy to evaluate the endometrial cavity for pathology.

It is contraindicated in patients with known or suspected pelvic infection. Risks include haemorrhage uterine perforation, infection, and Asherman syndrome.

### 4.4. Resectoscopic Endometrial Ablation Techniques

Transcervical resection of the endometrium (TCRE) has been considered the criterion standard cure for menorrhagia for many years [31]. This procedure requires the use of a resectoscope (i.e., hysteroscope with a heated wire loop), and it requires time and skill. The primary risk is uterine perforation.

Roller-ball endometrial ablation essentially is the same as (TCRE), except that a heated roller ball is used to destroy the endometrium (instead of the wire loop).

It has the same requirements, risks, and outcome success as TCRE. Satisfaction rates are also equal to those of TCRE [32].

Endometrial laser ablation requires Nd : YAG equipment and optical fiber delivery system.

The laser is inserted into the uterus through the hysteroscope while transmitting energy through the distending media to warm and eventually coagulate the endometrial tissue.

Disadvantages include the high expense of the equipment, the protracted time required to do the procedure, and the risk of excessive fluid uptake from the distending media infusion and irrigating fluid.

This technique has largely been replaced by the nonresectoscopic systems (discussed below).

Radiofrequency electricity is a detailed microprocessor-based unit with a bipolar gold mesh electrode array. It contains a system for determining uterine integrity based upon the injection of CO_2_.

The device is placed transcervically, the array is opened, and electrical energy is applied for 80 to 90 seconds, desiccating the endometrium.

Balloon thermohydrotubation is similar to the other procedures above as the aim is to destroy the endometrial lining. Heated fluid is instilled into the uterine cavity via a balloon. This coagulates the endometrium and this invariably stops the menorrhagia. The procedure appears to be simpler than many of the others mentioned with less side effect and similar efficacy.

## 5. Surgical Techniques

### 5.1. Myomectomy

Myomectomy can be useful in women who wish to retain their uterus and/or fertility.

Since myomectomy can be associated with significant blood loss, this procedure is often reserved for cases of a single or few myomas. In skilled hands many fibroids can be removed with the use of a hemostatic agent. The procedure of choice is the use on vasopressin injected perivascularly around the uterine and ovarian vessels. This occludes both arms of the anastamosis and hence this has been found to be superior to older methods such as tourniquets which occlude only the uterine vessels.

The use of vasopressin is relatively new and still evolving. The drug is usually diluted to 1 : 19 or 1 : 49 mls normal saline if there are many fibroids. Both sides of the broad ligament are injected to form a bleb around the vessels. Great care must be taken to avoid intravascular injection as this can cause systemic vasoconstriction with cardiac ischemia, right-side cardiac venous return overload, and left-side arterial constriction with acute hypertension and left hear failure.

Myomectomy is usually achieved with lowered blood loss; however, the cavities must be closed off securely to avoid secondary haemorrhage [33].

Great care must be taken in handling the tissue and placement of as few incisions as possible to avoid tubal occlusion form direct damage or postoperative adhesions.

The use of postoperative adhesion barriers in this era is mandatory but this needs further study.

### 5.2. Hysterectomy

Hysterectomy provides definitive cure for fibroids.

This procedure is more expensive and results in greater morbidity than ablative procedures.

A study by Roberts et al. [34] reviewed the cost effectiveness of first-generation and second-generation endometrial ablative techniques, hysterectomy, and the levonorgestrel-releasing intrauterine system (Mirena) for the treatment of heavy menstrual bleeding [34]. Although the authors did not define “heavy menstrual bleeding,” their analysis concluded that the most cost-effective initial treatment for menorrhagia that yielded the best quality of life was hysterectomy.

**Other Techniques**

Hysterectomy has been found to be safer than myomectomy as there is less bleeding; however, this is a definitive procedure removing any further reproductive desires for most women. Hysterectomy is also associated with other complications such as bladder or ureteric injury and also bowel injury. The risk of posthysterectomy vault prolapsed is a well-known entity, and this has resulted in many gynaecologists especially in Europe doing subtotal hysterectomies in order to avoid damaging the supports of the vagina. Others claim that removal of the cervix is more risky with more likelihood of damage to the urinary tract. However, in countries where cancer of the cervix is more common it is recommended that the cervix be removed since post hysterectomy cancer of the cervix is more difficult to treat.

Reattaching the cardinal ligaments to the vaginal vault after total hysterectomy seems to work well in our setting as vaginal vault prolapsed is not as common in our patients with this procedure.

### 5.3. Uterine Artery Embolisation

Uterine artery embolization (UAE) is a procedure where an interventional radiologist uses a catheter to deliver small particles that block the blood supply to the uterine body. The blood supply to the fibroids is said to be more tenuous than the uterus so the end result is that the fibroids become necrosed and shrink. The procedure works well in many women who do not want surgery. However, it can sometimes diminish fertility as the endometrium and myometrium can also be necrosed. Its effects on future fertility need further evaluation in large studies [35].

## 6. Summary

Uterine fibroids are very common in all ethnicities. They are especially problematic in women of Afro-Caribbean ancestry. Many women need no intervention for their fibroids. Many women only need conservative treatment. This can be medical treatment or surgical. The management of uterine fibroids requires the balance of the complications of the fibroids versus the risks of the treatment options.
